# Transcriptomic and proteomic studies of condylar ossification of the temporomandibular joint in porcine embryos

**DOI:** 10.1002/ame2.12326

**Published:** 2023-05-31

**Authors:** Lei Xiang, Yongfeng Li, Xuewen Wang, HuaWei Liu, Ping Chang, Xiaodan Mu, Tengyue Tianteng, Min Hu

**Affiliations:** ^1^ Beijing Research Institute of Traumatology and Orthopaedics Beijing China; ^2^ Department of Stomatology Beijing Friendship Hospital, Capital Medical University Beijing China; ^3^ Institute for Laboratory Animal Resources National Institutes for Food and Drug Control Beijing China; ^4^ Department of Stomatology the First Medical Center of PLA General Hospital Beijing China; ^5^ State Key Laboratory of West China College of Stomatology Sichuan University Cheng Du China

**Keywords:** condyle, embryo, miniature pig, ossification, proteomics, transcriptome

## Abstract

**Background:**

The ossification mechanism of the temporomandibular joint (TMJ) condyle remains unclear in human embryo. The size and structure of TMJ, shape of articular disc and the characteristics of omnivorous chewing in the pig are similar to those of humans. The pig is an ideal animal for studying the mechanism of ossification of the TMJ condyle during the embryonic period.

**Method:**

In a previous study by our group, it was found that there was no condylar ossification on embryonic day(E) 45, but the ossification of condyle occurred between E75 and E90. In this study, a total of 12 miniature pig embryos on E45 and E85 were used. Six embryos were used for tissue sections (3 in each group). The remaining six embryos were used for transcriptomic and proteomic studies to find differential genes and proteins. The differentially expressed genes in transcriptome and proteomic analysis were verified by QPCR.

**Results:**

In total, 1592 differential genes comprising 1086 up‐regulated genes and 506 down‐regulated genes were screened for fold changes of ≥2 to ≤0.5 between E45 and E85. In the total of 4613 proteins detected by proteomic analysis, there were 419 differential proteins including 313 up‐regulated proteins and 106 down‐regulated proteins screened for fold changes of ≥2 to ≤0.5 between E45 and E85. A total of 36 differential genes differing in both transcriptome and proteome analysis were found. QPCR analysis showed that 14 of 15 selected genes were consistent with transcriptome analysis.

**Conclusion:**

Condylar transcriptome and proteomic analysis during the development of TMJ in miniature pigs revealed the regulatory genes/proteins of condylar ossification.

## Graphical abstract

In this study, 15 differential genes which associated with bone development, regeneration and pathogenesis of osteoarthritis through literature reading were selected for QPCR verification. The up‐regulation or down‐regulation trend of 14 candidate genes was consistent with the results of transcriptome sequencing. The expression of AFP gene was too low not to be detected on E85.

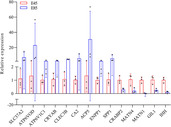

## 1 INTRODUCTION

The temporomandibular joint (TMJ) is a synovial, double, and bicondylar joint, which is composed of mandibular condyle, articular disc, articular fossa, superior and inferior articular cavities and associated muscles and tendons.^1^, ^2^ The mandibular condyle which is mainly formed by endochondral ossification is the growth and development center of the mandible. The condyle plays an important role in the development of the cranio‐maxillofacial region, with abnormal development leading to underdevelopment or overdevelopment of the mandible. Its growth and development consists of the proliferation, maturation, ossification and new bone deposition of the condylar cartilage.^3^ The TMJ condylar cartilage is different from the cartilage of long bone joint. It is secondary cartilage affected by both local factors and growth factors. The mechanism of secondary cartilage growth and cartilage formation depends largely on the external environment. The cell source of condylar cartilage is also different from that of long bone articular cartilage. TMJ condylar cartilage is fibrocartilage, developed from embryonic ectoderm and characterized by type I collagen, while long bone joint cartilage comes from the mesoderm and is hyaline cartilage, mainly type II collagen and mucopolysaccharide.^4^, ^5^

Studies of embryonic morphological development of TMJ began as early as 1952, but studies of the ossification mechanism of TMJ condylar cartilage lagged behind. There are few studies on the molecular regulation mechanism of TMJ development in the embryonic stage. These studies were based on rodents. Some classical signal pathways and transcription factors were discovered such as Wnt/ß‐catenin, IHH, TGF‐ß, SOX9 and RUNX2.^6^, ^7^, ^8^, ^9^, ^10^, ^11^, ^12^, ^13^, ^14^, ^15^ However, there are great differences in TMJ structure between rodents and humans. For example, compared with humans, TMJ articular fossa in rodents is shallow and flat, there are no articular nodules, the volume and function of the lateral pterygoid muscle are smaller, in the process of development the superior articular cavity is formed first, followed by the inferior articular cavity, and the TMJ articular disc rarely becomes fibrocartilage with increasing age. These differences have undoubtedly delayed progress in TMJ‐related research.^16^ In recent years, the whole genome of the pig has been sequenced. It has been found that the genomic composition of the pig is highly homologous to that of humans and the physiological and anatomical characteristics of pig are very similar. For example, the size, structure of TMJ, shape of the articular disc and characteristics of omnivorous chewing are very similar to those of humans. Pig epiblast stem cells are excellent models for discovering the properties of human pluripotent stem cells. Chondrogenesis of the TMJ originates from the ectoderm and pig ectodermal stem cells can mimic human ectodermal cell development.^17^ As a model animal for TMJ studies, the pig has obvious advantages over rodents (rat and mice), carnivores (dog and cat) and herbivores (cattle, sheep and rabbits).^18^ However, the signal molecules and transcription factors related to chondroossification in porcine TMJ embryo development have not been reported.

In previous experiments of this study, involving histological analysis of pig TMJ development at embryonic days (E) 35, E45, E55, E75, E90 and postnatal day (P) 1, it was found that day E45 and the days between E75 and E90 were the key periods of condylar cartilage development and ossification.^19^ In this study, transcriptome and proteomic analysis were used to find the key regulatory factors of condylar ossification and the signal pathways related to condylar cartilage differentiation.

## 2 METHODS

The experiments were conducted according to the guidelines of the Ethical Review of Laboratory Animal Welfare (GB/T358922018). The study was approved and supervised by the Institutional Animal Care and Use Committee of Chinese PLA General Hospital (Beijing, China; approval document no. 2020‐X16‐109). Twelve embryos of Bama miniature pigs on E45 and E85 were purchased from Beijing Kuibu Shichuang Biotechnology Co., Ltd (license number: SCK2018‐0011).

### 2.1 Histological staining

The embryos were dissected to fully expose the TMJ. The whole TMJ (including condyle, articular disc, articular fossa, superior and inferior articular cavities) was obtained from the zygomatic arch to the mandible. Twelve TMJs obtained from E45 and E85 embryos (*n* = 3 for each group) were fixed in 10% formalin for 2 days. E85 embryos (*n* = 3) were decalcified in 0.5 mol L^−1^ ethylenediaminetetraacetic acid solution (Solarbio Biological Company) for 14 days and rinsed with tap water for 10 min. Temporomandibular joint tissues from E45 and E85 embryos were embedded in paraffin for analysis. Semi‐serial sagittal sections (3 μm) of the TMJ were prepared using a microtome (HM 325; Thermo Fisher Scientific, Waltham, MA, USA). The samples were stained with Hematoxylin–Eosin (HE) and Safranin O‐Fast green for analysis. The HE staining was performed using a Leica AutoStainer XL (Leica Biosystems, Wetzlar, Germany). Safranin O‐Fast green staining was performed following the instructions for the kit. The iview6.3.6 software was used to analyze the tissue sections.

### 2.2 Transcriptome and proteomic analysis

Twelve TMJs were obtained from E45 and E85 embryos (*n* = 3 for each group). The condyle was excised between the lowest point of the sigmoid notch and the highest point of the condyle. The condyle sample from each embryo was divided into two parts. The first portion of tissue was used to extract RNA (RNeasy Mini Kit, Cat#74106, Qiagen), construct RNA library and Illumina sequencing (Shanghai Huaying Biopharmaceutical Technology Co., Ltd). Genomic mapping analysis of the sequencing result was performed using HISAT2 software. The reference genome version is Sus_scrofa.Sscrofa10. The second portion of tissue was used for protein extraction. The protein samples obtained were redissolved, subjected to reductive alkylation, enzymatic hydrolysis, desalting and tandem mass spectrometry. The proteome Discoverer 2.4 software was used to search the database to identify the proteome. Analysis of protein expression used the relative quantitative method of Label free (Shanghai Huaying Biopharmaceutical Technology Co., Ltd.). Student's t test was used to calculate the *p* value of the two groups. The intergroup ratio of fold changes ≥2 or ≤0.5 was selected as indicating for differential gene/protein. The genes with differences in transcriptome and proteology levels were screened as candidate genes. Differential genes/proteins between groups were analyzed by GO analysis and KEGG analysis.

### 2.3 Verification of differential genes

Fifteen different genes were selected for QPCR verification. The existing literature shows that these 15 differential genes were associated with bone development, regeneration and pathogenesis of osteoarthritis. They may also be associated with ossification of the temporomandibular joint condyle during the embryonic stage. Total RNA was extracted (RNeasy Mini Kit, Qiagen) from condylar tissue. The RNA was reverse transcribed into cDNA using the PrimeScriptTM RT regent Kit with gDNA Eraser (Takara, RR047A). The mRNA sequence information of the 15 candidate genes was downloaded from NCBI, and the PCR primers were designed using the BLAST module in the NCBI database (Table 1). The reaction system consisted of NovoStart SYBR High‐Sensitivity QPCR SuperMix 5 ul, F 0.3 ul, R 0.3 ul, cDNA 0.3 ul, and RNase‐free water 4.1 ul. The reaction incubation sequence was 95°C for 1 min, 95°C for 20 s, 60°C for 20 s, 72°C for 30 s for 45 cycles, and 72°C for 5 min.

**TABLE 1:** The QPCR primer sequence.

Gene name	Primer sequence (F)	Primer sequence (R)
SLC37A2‐132 bp	GTTCCGAGGCTTCATCCTGTT	GTGTTGTTGGTGGGCTGGAT
ACP5‐181 bp	CCCATCCTGCGTTTTGTGG	TGTCTTTGGCATCATGCACC
ATP6V0D2‐155 bp	GCGCTGAGCTGTACTTCAATG	GCCGTAATCAGTGGTCTGGAG
ATP6V1C1‐138 bp	ACCTGTCAGCAAACATGGGA	AGCCAGTTCATCCGACAAACC
CRYAB‐137 bp	GGATTGACACTGGGCTCTCA	TTCATGTTTGCCGTGCACCT
IHH‐176 bp	TTCAAGGACGAGGAGAACACC	CGGCCTTCATAATGCAGCGA
CA2‐175 bp	GCCAGTCCCCTGTTGACATC	GCAAGAGGTCCACCTTCCAG
ENPP1‐143 bp	GGAGGAGCAGCTGGAGAAG	CTTTGGCACAGCTTGGTTTCA
SPP1‐131 bp	CAGTGATAGCCTTCTGCCTCT	GTCAGGCTTTAGCAATGTGGC
AFP‐145 bp	GGATTCTTCCCAATGTTCTGC	ACTGCCAGTGGATTTCTCAAT
MATN4‐192 bp	CCAGCCCCCGGGTTAAAT	GGGAACAGAGAGCGGAACTA
CRABP2‐145 bp	CGATCGGAAAACTTCGAGGA	GCACGGTGGTAGAGGTTTTGA
MATN1‐145 bp	CCTTAAGTGATGCCGAGGGT	CCGATGGCGAACAGCTCAAT
GIL1‐142 bp	AGAGAGACCAACAGCTGCAC	TGGGGGAGGTTCGGATAACT
CLEC3B‐130 bp	GAGCTTTGGGGGCCCTACTT	CAAGTATCTTTGGGCTTGCGG

## 3 RESULTS

### 3.1 Histological staining

**FIGURE 1:**
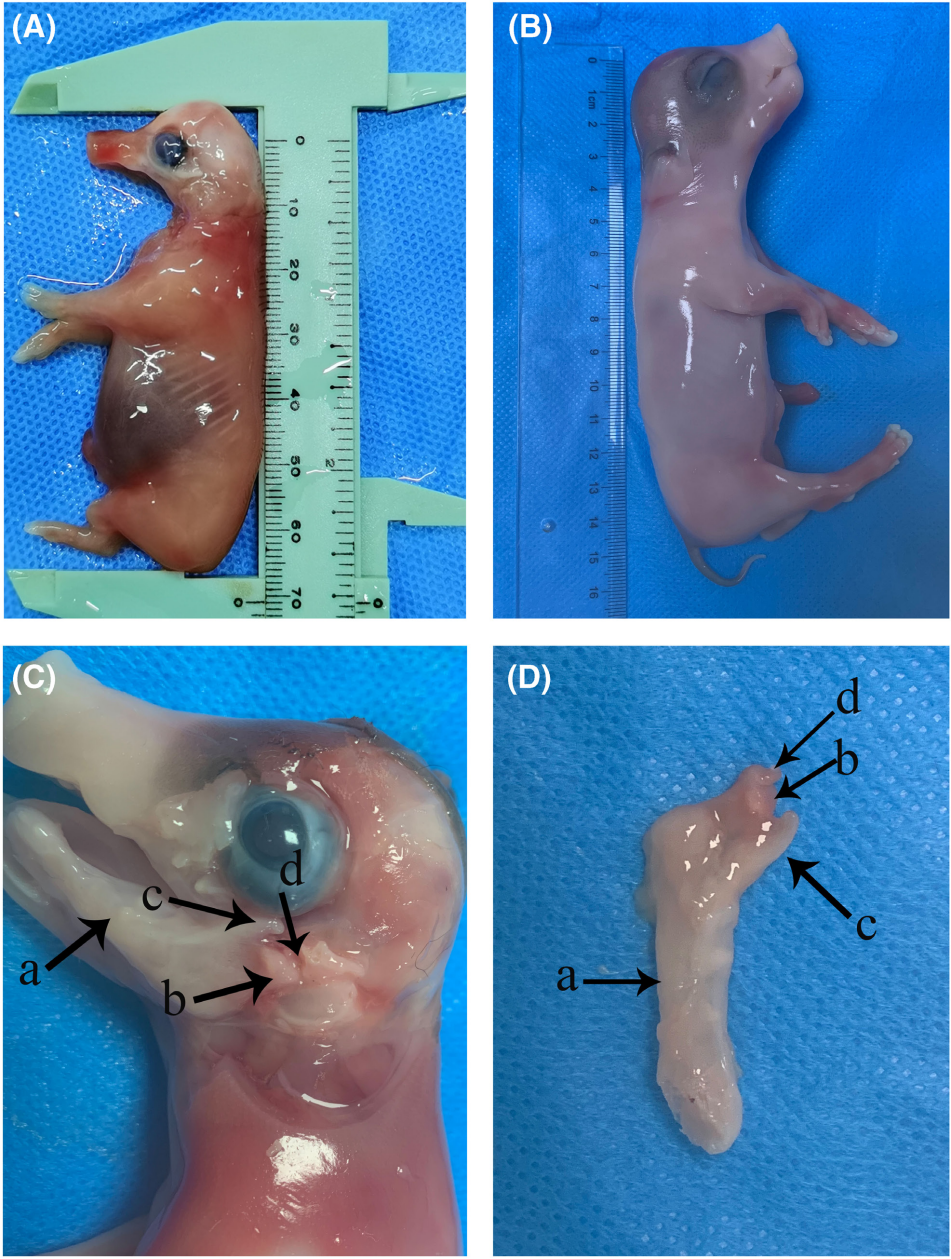
Anatomical characteristics of miniature pig temporomandibular joints (TMJs) at embryonic day (E) 45 (removal mandible) and E85. (A) Crown‐rump lengths (CRLs) at E45. (B) Crown‐rump lengths (CRLs) at E85. (C) Anatomy of TMJ at E85: a. mandible bone; b. condyle; c. coracoid process; d. articular fossa. (D) structural anatomy of mandible: (a) mandible bone; (b) condyle; (c) coracoid process; (d) articular disc.

**FIGURE 2:**
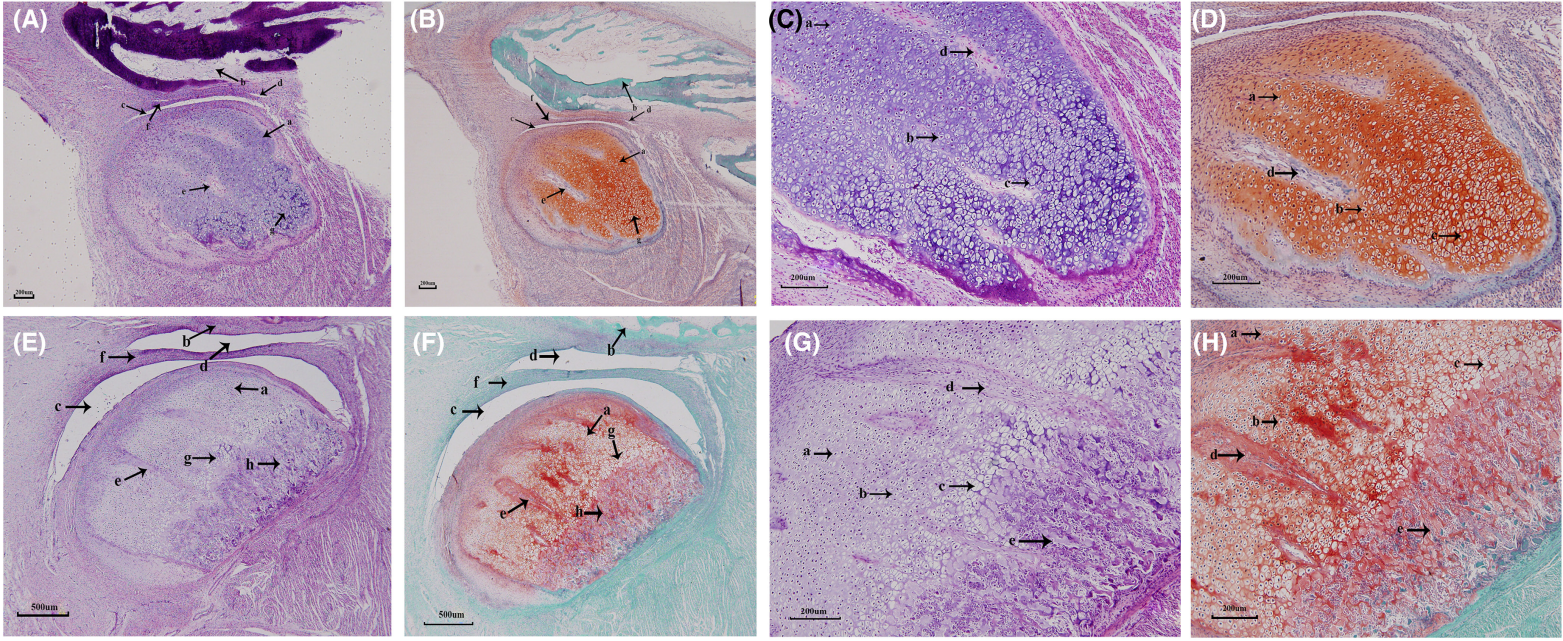
Hematoxylin–Eosin and Safranin O‐Fast green staining of TMJ at embryonic day (E) 45 and E85 in miniature pigs. (A–D) TMJ sagittal Hematoxylin–Eosin and Safranin O‐Fast green staining on E45. (A,B) a. Condylar chondrocytes;b. temporal bone; c. inferior articular cavity; d. superior articular cavity; e. bone marrow cavity; f. articular disc; g. hypertrophic chondrocyte layer; (C,D) a. prechondral cell; b. Chondrocytes; c. hypertrophic chondrocytes; d. bone marrow cavity. (E–H) TMJ sagittal Hematoxylin–eosin and Safranin O‐Fast green staining on E85. (E,F) a. Condylar chondrocytes; b. temporal bone; c. inferior articular cavity; d.superior articular cavity; e. bone marrow cavity; f. articular disc; g. hypertrophic chondrocyte layer;h. mineralized bone tissue. (G,H) a. prechondral cell; b. Chondrocytes; c. hypertrophic chondrocytes; d. bone marrow cavity; e. mineralized bone tissue.

The crown‐rump lengths (CRL) of E45 and E85 embryos were respectively 6.5 cm and 14.5 cm. The complete TMJ structures including condyle, articular disc, articular fossa, superior and inferior articular cavities were observed by histological anatomy (Figure 1). Histological staining showed that the condyle is separated from the temporal bone (Figure 2A,B,a,b) at E45. The articular disc is not fully developed (Figure 2A,B,f) at this time. The inferior articular cavity is formed (Figure 2A,B,c), but the superior articular cavity is not fully formed – only cracks are visible at both ends of the articular disc (Figure 2A,B,d). The condyle is occupied by a large number of chondrocytes. It can be seen that the prechondrocytes are round or fusiform (Figure 2C,D,a) and the chondrocytes are oval in shape (Figure 2C,D,b). A small number of hypertrophic chondrocytes are vacuolar (Figure 2C,D,c). No mineralized bone tissue formation was found in the whole condylar structure at E45. By the 85th day of pregnancy, the inferior articular cavity, superior articular cavity (Figure 2E, Fc,d) and the articular disc (Figure 2E,F,f) have been formed. A large number of hypertrophic chondrocytes (Figure 2E,F,g and G,H,c) and mineralized bone tissue (Figure 2E,F,h and G,H,e) can be seen at the base of the condyle.

### 3.2 Transcriptome analysis

**FIGURE 3:**
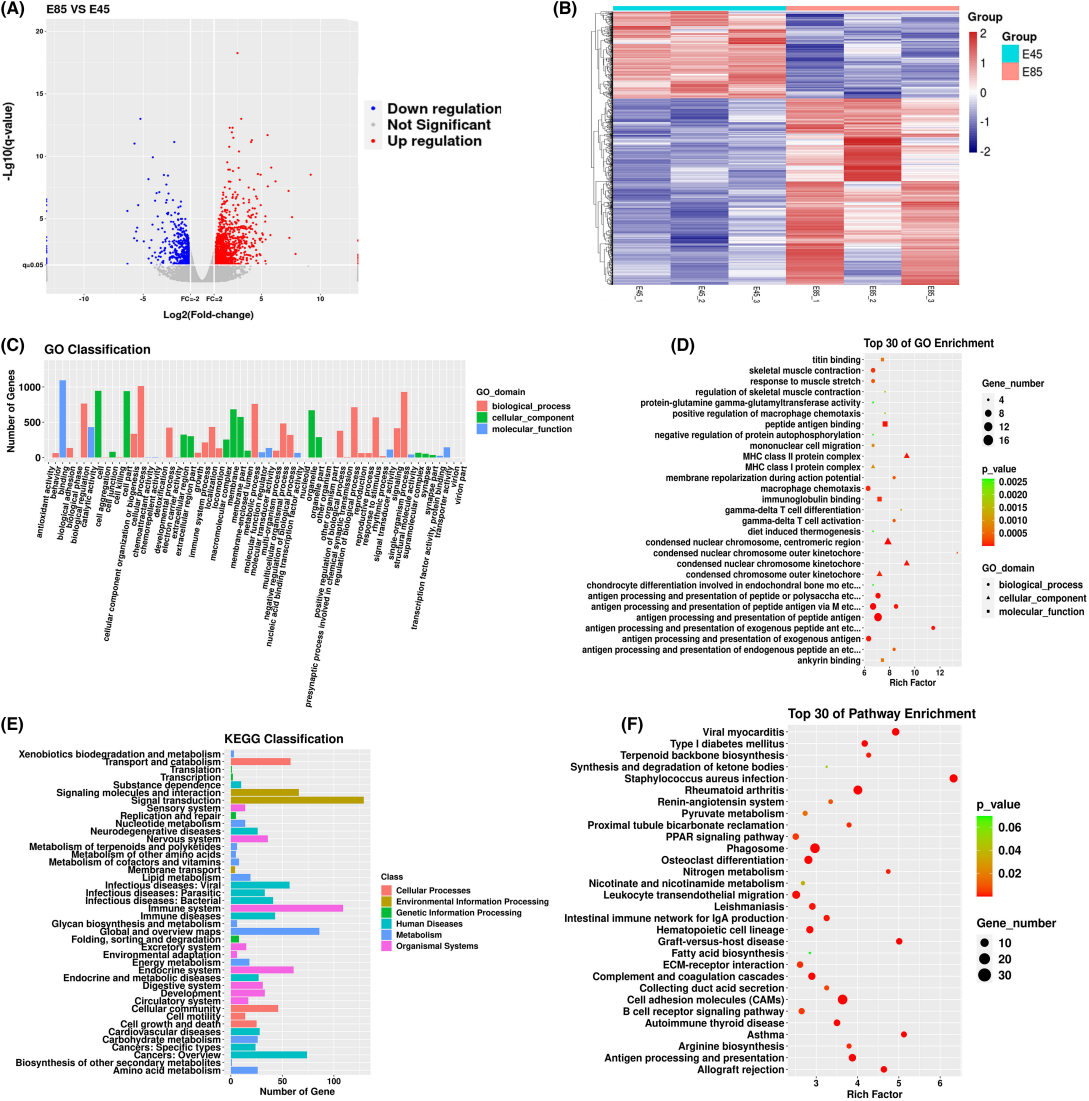
Transcriptional analysis of condylar tissue at embryonic day (E) 85 and E45. (A) Analysis of differential gene volcano map. Red, upregulated differential genes; blue, downregulated differential genes; (B) Differential gene cluster analysis map. Red, high expression; blue, low expression. Rows represent genes, columns represent sample, and color indicates the level of expression; (C) GO classification statistics of differential genes; (D) GO enrichment analysis of differential genes the top 30 enriched genes. The ordinate is the name of the specific GO entry. The color of the dot indicates the Q value of GO, the shape of the dot indicates that the corresponding GO entry belongs to the three categories of GO database, and the size of the dot indicates the number of genes mapped to a GO entry. (E) Pathway classification statistics of differential genes; (F) Pathway enrichment analysis of differential genes.

The sequencing results of the E85 and E45 condylar tissue transcript groups were to analyze porcine genomic mapping using HISAT2 software. A total of 25 322 genes were detected, and 19 584 differential genes between E85 and E45 were found. Of these, 1592 genes were consistent with intergroup ratio fold changes of ≥2 and ≤0.5. These genes were screened and a volcanic map was drawn (Figure 3A). Among the 1592 differential genes, 1086 genes were upregulated at E85 compared with E45. A total of 506 genes were downregulated (the top 15 genes with upregulation and downregulation differences are shown in Table 2). The genes with similar expression patterns are clustered together to draw the cluster analysis map of different genes (Figure 3B).

**TABLE 2:** The top 15 differentially expressed genes between E85 and E45.

Gene ID	Gene name	Log2FC	*p* value	*Q* value	Up/down
ENSSSCG00000010947	FBP2	9.173271	3.77E‐12	2.95E‐09	UP
ENSSSCG00000013730	KLF1	7.303407	1.20E‐10	5.88E‐08	UP
ENSSSCG00000007260	BPIFA1	−6.3363282	1.11E‐08	2.36E‐06	DOWN
ENSSSCG00000028347	PPP1R11	−5.765921145	6.53E‐06	0.000361392	DOWN
ENSSSCG00000026945	LRRC2	5.762317	2.95E‐06	0.000203	UP
ENSSSCG00000003781	ERICH3	−5.736130847	6.32E‐15	9.52E‐12	DOWN
ENSSSCG00000011034	ST8SIA6	−5.601663427	1.31E‐06	0.000106052	DOWN
ENSSSCG00000007507	PCK1	5.544021	4.53E‐11	2.53E‐08	UP
ENSSSCG00000022554	MATN1	−5.523017913	1.17E‐05	0.00056852	DOWN
ENSSSCG00000015663	C4BPA	5.522999478	8.16E‐16	2.00E‐12	UP
ENSSSCG00000022554	MATN1	−5.523017913	1.17E‐05	0.00056852	DOWN
ENSSSCG00000017313	CRHR1	−5.506249529	5.02E‐07	5.14E‐05	DOWN
ENSSSCG00000017444	KRT15	−5.422513097	3.26E‐09	8.09E‐07	DOWN
ENSSSCG00000018084	ND3	5.382714654	0.003817619	0.043708726	UP
ENSSSCG00000008648	RSAD2	5.356808108	8.59E‐06	0.000453618	UP

Abbreviations: Gene ID, Gene ID in the database; Gene name, Official name of gene; Log2FC, The logarithm of the multiple of difference; *p* value, *p* value of significance test; *Q* value, Correction value of *p* value; Up/Down, Up‐or down‐regulation.

The differential genes were classified by GO analysis as cell component (CC), molecular function (MF) and biological process (BP) (Figure 3C). The CC were mainly concentrated in cells and cell components category. BP were mainly concentrated in biological regulation, cellular process, single organism process, metabolic process and biological process regulation categories. MF mainly focused on binding and catalytic activity. The GO items with *p* < 0.05 were selected as significantly enriched GO items (Figure 3D). The differential genes were mainly enriched in polypeptide antigen binding (GO:0042605, GO:0002495, GO:0002474) in MF, antigen processing and presentation (GO:0048002) of polypeptide antigen in BP, and concentrated nuclear chromosome and centromere region (GO:0000780) in CC.

Similar to the GO classification statistics, the number of differentially expressed genes in each pathway category of KEGG was counted (Figure 3E). The differential genes were mainly concentrated in the signal transduction, metabolism and immune system categories. The differentially expressed genes were analyzed by pathway using the Fisher precise test. *p* values were obtained using the Fisher accurate test calculation. Pathway with *p* < 0.05 values indicated a significantly enriched pathway (Figure 3F). The KEGG enrichment analysis showed that differential genes were mainly enriched in cell phagosome (ssc04145), cell adhesion factor (ssc04514), rheumatoid arthritis pathway (ssc05323) and osteoclast differentiation (ssc04380) categories.

### 3.3 Proteomic analysis

A total of 4613 proteins were obtained from E45 and E85 condylar tissue by protein spectrum analysis. The normalized signal mean of all samples in the two groups was calculated. The inter‐group ratio fold change was calculated, and Student's t test was used to calculate the *p* value of the two groups. Proteins with ratios of fold changes of ≥2 and ≤0.5 were regarded as differential proteins. There were 419 differential proteins including 313 proteins that were upregulated between E85 and E45 and 106 proteins that were downregulated. The top 15 differential proteins expressed are shown in Table 3.

**TABLE 3:** The top 15 differentially expressed proteins at E85 and E45.

Accession	Gene Symbol	MW [kDa]	Ratio	*p* value	Up/Down
A0A5G2R0J2	MORF4L1	26.7	362.51933	1.5192E‐06	Up
A0A5G2QXK1	SLC37A2	53	46.49671	0.029805699	Up
A0A287A7H6	DDX47	54.9	0.02698	7.4986E‐06	Down
A0A287BR31	MICAL3	230.7	0.02946	0.020885658	Down
F1SNG6	EXOC4	110.5	30.96372	1.81996E‐05	Up
F1RP64	SLITRK1	77.5	28.59457	0	Up
Q6J1I8	RNF114	25.6	26.0303	0.000612051	Up
A0A287AQR8	HSPB6	17.4	17.10203	0.02856692	Up
I3LTQ6	SFXN3	34.6	0.05923	0.019914593	Down
A0A5G2QWF0	ATP6V0C	15.6	16.08524	0.031679372	Up
A0A5S8KUU6	ACP5	41.4	15.33802	0.037798749	Up
P02587	TNNC2	18	14.30943	0.000396968	Up
A0A287AB08	FNTA	42.7	0.07926	0.026714666	Down
A0A287AVF3	ENO2	44.7	0.08501	0.007606717	Down
A0A5G2R214	IFI30	48.4	9.25	11.61868	Up

Abbreviations: Accession, protein landing number; Gene Symbol, gene name; MW [kDa], molecular weight of protein; Ratio, E85/E45 comparison ratio value; *p* value, E85/E45 comparison *p* value value; Up/down, up or down regulation relationship.

**FIGURE 4:**
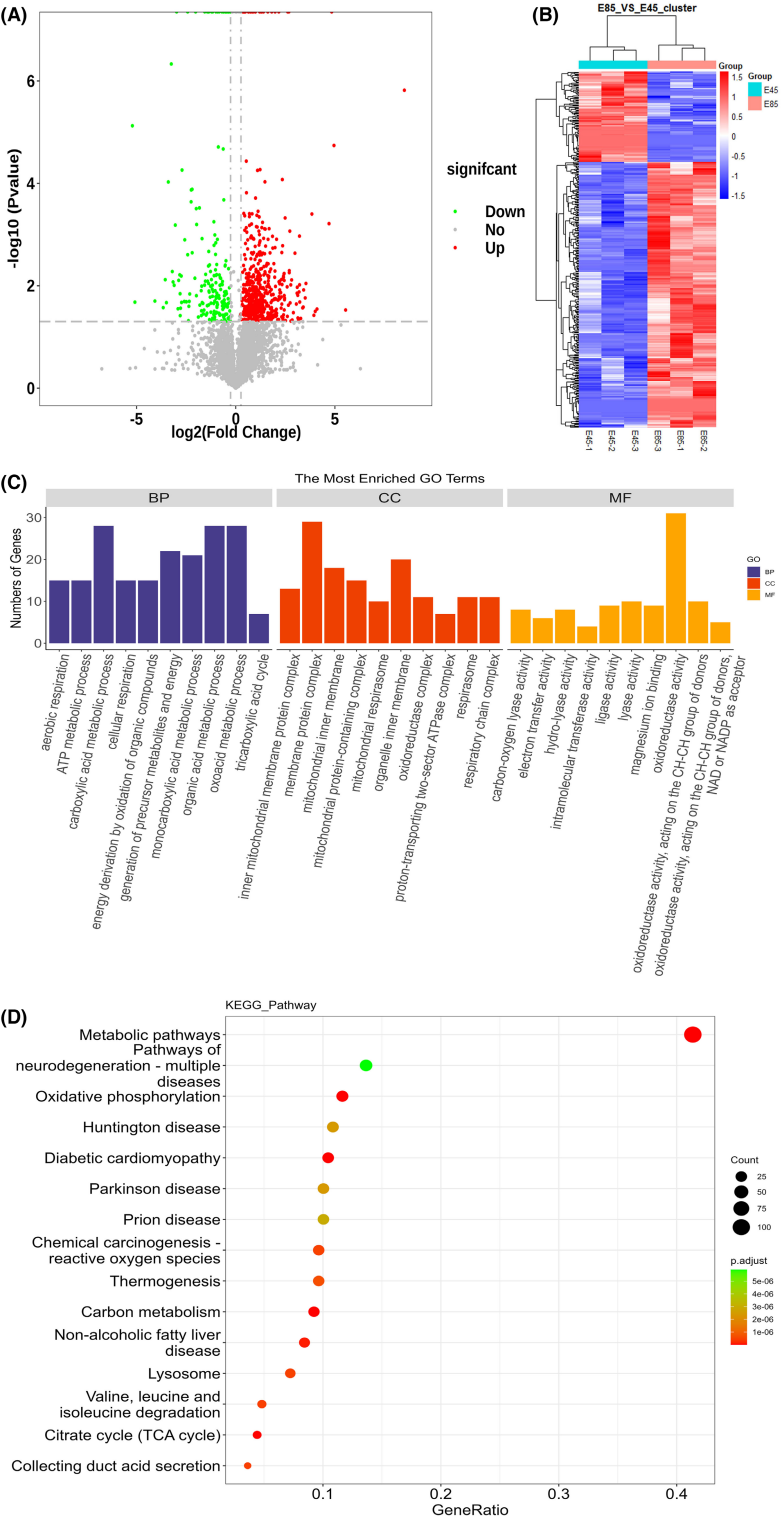
Proteomic analysis of condylar tissue at embryonic day (E) 85 and E45. (A) Differential protein volcano map. The ordinate is ‐log10 (*p* value) value, and the abscissa is log2 (fold change) value. Red dots, up‐regulated differential proteins; green dots, down‐regulated differential proteins. Other proteins are represented by red and black dots. (B) Differential protein cluster analysis map. The abscissa represents the sample name between the groups, the ordinate represents the differential genes, red represents a high expression value of the differential genes in the grouping sample, and blue represents a low expression value of the differential protein in the grouping sample. (C) GO enrichment analysis of differential proteins. The vertical axis is numbers of genes, and the horizontal axis is the entry. (D) KEGG Pathway analysis. The horizontal axis is the GeneRatio. The vertical axis is the entry. The color of the points indidcates the *p* value. The size of the point indicates the number of genes.

A volcanic diagram and a cluster analysis map of the 419 differential proteins were drawn (Figure 4A,B), and the 10 items with the smallest *p* values were selected to draw Barplot map (less than 10 items not shown, Figure 4C). GO enrichment analysis of differential proteins before and after condylar ossification showed that differential proteins were mainly enriched in the process of small molecule metabolism in BP, cytoplasm in CC, and catalytic activity and ion binding in MF. KEEG pathway enrichment analysis (Figure 4D) showed that the differential proteins were mainly concentrated in the metabolic pathway (ssc01100) and oxidative phosphorylation pathway (ssc00190) categories.

### 3.4 Cross‐analysis in transcriptome and proteomics

Thirty‐seven differential genes including 29 upregulated genes/proteins and 8 downregulated genes/proteins differing in both the transcriptome and proteomic analysis were found. It is interesting that the expression of IHH and TNMD genes was upregulated in the proteomics, but downregulated in transcriptome. The results of the cross‐analysis of transcriptome and proteomics results are shown in Table 4.

**TABLE 4:** Genes differing in transcriptome and proteome analysis.

Gene ID	Gene name	Protein accession	Log2FC (Genen)	Ratio (protein)	UP or Down (gene/protein)
ENSSSCG00000022536	SLC37A2	A0A5G2QXK1	2.59853204	46.49671	UP/UP
ENSSSCG00000013612	ACP5	A0A5S8KUU6	3.743463467	15.33802	UP/UP
ENSSSCG00000007424	TNNC2	P02587	2.618041726	14.30943	UP/UP
ENSSSCG00000013901	IFI30	A0A5G2R214	1.950977888	11.61868	UP/UP
ENSSSCG00000010281	PSAP	P81405	1.205685019	10.72283	UP/UP
ENSSSCG00000006138	ATP6V0D2	F1RXD2	3.298755158	10.58796	UP/UP
ENSSSCG00000006054	ATP6V1C1	F1S0R4	1.076461102	9.2707	UP/UP
ENSSSCG00000000866	MYBPC1	A0A5G2QM90	2.400549978	6.14714	UP/UP
ENSSSCG00000015025	CRYAB	Q7M2W6	1.141693331	5.70188	UP/UP
ENSSSCG00000010359	LDB3	A0A287A435	2.114918964	5.42663	UP/UP
ENSSSCG00000026098	CMYA5	I3LPD6	3.449631388	4.13448	UP/UP
ENSSSCG00000005316	TPM2	A0A287AN33	1.748847215	4.0381	UP/UP
ENSSSCG00000016204	IHH	F1SRW6	−3.928972339	3.88659	DOWN//UP
ENSSSCG00000008982	STBD1	A0A5G2R9Y3	1.327886745	3.4938	UP/UP
ENSSSCG00000012480	TNMD	F1S1M5	−2.449170995	3.47327	DOWN//UP
ENSSSCG00000013022	PYGM	A0A287B6I2	2.388427861	3.07033	UP/UP
ENSSSCG00000009111	SYNPO2	A0A286ZLL9	1.847381108	3.05036	UP/UP
ENSSSCG00000006140	CA2	A0A287B6M0	2.277848499	2.85439	UP/UP
ENSSSCG00000011299	CLEC3B	F1SRC8	2.2829262	2.74049	UP/UP
ENSSSCG00000010281	PSAP	A0A287A0D2	1.205685019	2.66323	UP/UP
ENSSSCG00000016720	PGAM2	B5KJG2	2.029791498	2.64612	UP/UP
ENSSSCG00000004193	ENPP1	A0A287AT99	1.043508529	2.6114	UP/UP
ENSSSCG00000009216	SPP1	P14287	2.032302041	2.49845	UP/UP
ENSSSCG00000010494	SORBS1	A0A286ZJT5	1.013649233	2.44681	UP/UP
ENSSSCG00000010123	CLDN5	C3VML1	1.158519528	2.34631	UP/UP
ENSSSCG00000006648	CTSS	F1SS93	1.109331713	2.33888	UP/UP
ENSSSCG00000007133	ACSS1	A0A287B7Q9	1.355670156	2.22863	UP/UP
ENSSSCG00000017904	ENO3	Q1KYT0	2.716319039	2.20775	UP/UP
ENSSSCG00000008949	AFP	Q8MJ76	**–**	0.42722	DOWN/DOWN
ENSSSCG00000007391	MATN4	F1SDQ7	−3.360839174	0.39493	DOWN/DOWN
ENSSSCG00000016872	HMGCS1	A0A5G2QC33	−1.282129924	0.33675	DOWN/DOWN
ENSSSCG00000006472	CRABP2	F1RHI8	−1.627249104	0.30033	DOWN/DOWN
ENSSSCG00000011453	ITIH4	A0A287BGD6	1.443859596	0.27038	UP/UP
ENSSSCG00000022945	UCHL1	Q6SEG5	−1.574848989	0.25265	DOWN/DOWN
ENSSSCG00000022554	MATN1	I3L5Q7	−5.523017913	0.14355	DOWN/DOWN
ENSSSCG00000011640	TF	P09571	5.081498108	0.12091	UP/UP

*Note*: Log2FC, the multiple of transcriptional difference between E85 day and E45 day samples is logarithmic. Ratio, the ratio of the average relative expression of protein in E85 sample to that in E45 sample.

### 3.5 Quantitative real‐time PCR

**FIGURE 5:**
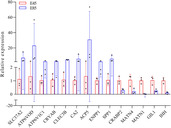
The 15 genes were selected and verified by QPCR. The upregulation or downregulation trend of 14 of the candidate genes was consistent with the results of transcriptome sequencing; the expression of the AFP gene was too low to be detected in the set of data at E85.

Fifteen differential genes were selected and verified by QPCR. The quantitative Real‐time PCR data analyzed according to the relative difference=2^−ΔΔCtq^ method, excel software analysis, Grphpad prism9 software drawing to show relative differences. The results showed that the upregulation or downregulation trends of 14 candidate genes were consistent with the results of transcriptome sequencing. However the expression of the AFP gene was too low to detect at E85. The consistency of the three technical repetitive samples in each group is good, but there are big differences among the three biological repetitive samples, and the error line is larger in the mapping (Figure 5).

## 4 DISCUSSION

The TMJ condylar cartilage and its subchondral bone formation is an important and understudied topic in dental research. The transition from chondrocytes to osteocytes in articular cartilage of long bone has been reported.^20^ There are still contradictory views on the biomolecular regulation mechanism that controls condylar cartilage replaced by bone tissue in the process of natural growth. At present, there are two main theories about the cause of cartilage osteogenesis. One explanation is that bone tissue is formed by programmed cell death (apoptosis) of hypertrophic chondrocytes, followed the MMp‐13 and MMP‐9 absorb the laterally mineralized matrix, finally potential bone marrow and blood vessel formed. The invading capillaries bring osteogenic progenitor cells and bone marrow stem cells that differentiate into osteoblasts.^20^, ^21^, ^22^ Another theory is that hypertrophic chondrocytes are transformed into osteoblasts without apoptosis.^23^ In this study, we used transcription and proteomics analysis to detect the molecular mechanism of condylar ossification in order to find the key regulatory molecules of condylar ossification.

In this study, histological observations showed that the condyle at E45 was occupied by a large number of chondrocytes, and scattered hypertrophic chondrocytes could be seen at the bottom of condyle, but no mineralized bone tissue was found. In contrast, at E85 a layer of mineralized bone tissue was formed at the base of the condyle. This difference in bone morphology is accomplished under a specific molecular regulation mechanism. In order to find these important ossification regulatory molecules, we used transcriptome analysis to identify 1592 differential genes (including 1086 upregulated and 506 downregulated genes) during the development of TMJ between E45 and E85. Among these differential genes, the expression of the top 15 genes was more than 32‐fold different between E45 and E85. (Table 2). The differential genes between E85 and E45 may play important roles in bone formation, but there are many biological processes such as splicing and modification in gene expression that prevent these genes from being translated into proteins to perform biological functions. Therefore, proteomic analysis was additionally performed in this study to more accurately identify target genes/proteins. In total, 419 differential proteins were found by proteomic analysis, and the expression of the top 15 differential proteins was more than 10 times ratio at E85 than at E45. (Table 3). Cross‐analysis of all the differential genes and proteins showed that there were a total of 37 differential genes (Table 4).

The molecular regulation mechanism of embryonic condyle ossification has not previously been reported. This preliminary study of the regulation mechanism of TMJ condyle ossification identified 37 genes which may play an important role in the formation of condylar osteoblasts, of which fifteen genes were verified by QPCR (Table 1). It was found that 14 genes were consistent with the transcriptome analysis results. These differential genes were mainly related to the formation and differentiation of osteoblasts, osteoclasts and chondrocytes.

Among the 14 differential genes, CRYAB, CLEC3B, ENPP1, SPP1, Gli1 and CRABP2 were mainly related to osteoblast differentiation, with the expression of CRABP2 showing a downregulation trend, while other genes showed an upregulation trend. In these six genes CRYAB, ENPP1, SPP1 and Gli1 participate in the differentiation of osteoblasts. CRYAB can promote Wnt signal transduction to regulate the osteogenic differentiation of bone marrow mesenchymal stem cells by ubiquitination and degradation of β‐catenin.^24^ The increase in CRYAB expression induces the bone marrow mesenchymal stem cells to differentiate into osteoblasts. Furthermore CRYAB plays an important role in chondrocyte proliferation and extracellular matrix formation. Elevated expression of CRYAB can also promote chondrocyte differentiation.^25^ ENPP1 can inhibit the deposition of hydroxyapatite (HA) by producing pyrophosphate (PPi), which plays an important role in regulating bone and soft tissue mineralization.^26^ Another gene involved in osteogenic differentiation is SPP1. It encodes osteopontin (OPN), which is secreted by osteoblasts and osteocytes in bone. Osteoblasts can induce OPN expression through the MAPK pathway, so that PPi and OPN cooperate with each other to inhibit the process of bone mineralization. High expression of SPP1 in bone regenerative medicine research can effectively increase osteogenesis.^27^ Gli1‐related studies have mainly focused on postnatal bone regeneration and bone homeostasis. Gli1 not only promotes osteoblast differentiation, but also inhibits osteoblast maturation to maintain normal bone homeostasis. Gli1^+^ cells gradually produce osteoblasts in all bone sites, and can mediate theTGF‐ß signaling pathway and play an important role in endochondral bone formation after fracture.^28^ Some studies found that Gli1^+^ cells possess mechanical stress, which may regulate changes in intracellular Ca^2+^ concentration through the inositol 1,4,5‐trisphosphate receptor (IP3R) to respond to mechanical force.^29^, ^30^ Compared with the above four genes, CLEC3B an CRABP2 have been less well studied in osteogenesis. CLEC3B is a major structural protein of the ocular lens and is abundant in the kidney, central nervous system, skeletal muscle and heart.^31^ In a study of osteoarthritis (OA), it was found that expression of CLEC3B in OA samples was much higher than that in normal articular cartilage, which may be related not only to osteoblasts, but also to the formation of OA.^32^, ^33^ CRABP2 may interact with LIMK1 and affect actin remodeling and osteogenic differentiation. Its mRNA and protein expression levels decreased during osteogenesis.^34^ In this study, it was found that the expression of CRYAB, CLEC3B, SPP1 and ENPP1 at E85 were significantly higher than that at E45 and the development of condyle, and the amount of mineralized bone tissue in E85 was also significantly higher than that in E45. Gli1 was significantly downregulated at E85, which was inconsistent with the results of postnatal Gli1 in osteoblast proliferation and differentiation. It may be that Gli1 may be regulated by different signal molecules in the embryonic stage than in the postnatal stage, and its regulatory role in the development of TMJ at the embryonic stage remains to be further researched.

In addition to genes related to osteogenesis, genes related to chondrocytes and osteoclasts were also found, such as Matn1, Matn4, IHH, SLC37A2, Acp5 and ATP6V0d2. Matn1, Matn4 and IHH are related to the proliferation and differentiation of chondrocytes. Matrilin proteins (Matn1, Matn2, Matn3 and Matn4) form the connective protein of cartilage extracellular matrix (ECM), which connects collagen II and proteoglycan. Although there was no abnormal bone formation in Matn1^−/−^ mice, the diameter of collagen fibers in cartilage increased, the mechanical transduction was abnormal, and the transcription levels of Acan and Col2a in chondrocytes decreased significantly. Matn4^−/−^ mice spontaneously developed osteoarthritis, accompanied by changes in the biomechanical properties of articular cartilage.^35^, ^36^ IHH is another gene associated with chondrocytes. It begins to express in mesenchymal condensates at E13.5d and is significantly enhanced at E15.5d in the TMJ condylar cartilage of mouse embryos.^7^, ^13^, ^14^ The downregulation of Matn1, Matn4 and IHH may be related to the continued osteogenesis and relative reduction of chondrocytes in the condyle during TMJ development. In our study, expression of Matn1 and Matn4 decreased significantly at E85, which may be related to the decrease in chondrocytes. Whether there are changes in the diameter of collagen fibers and biomechanical properties remain to be further confirmed. The SLC37A2, Acp5 and ATP6V0d2 genes are related to osteoclasts. These three genes were highly expressed at E85 compared with E45. These three genes in osteoclast formation have been reported. SLC37A1 and SLC37A2 are active in glucose‐6‐phosphate (G6P) exchange across endoplasmic reticulum during gluconeogenesis. Abnormal SLC37A2 causing bone dysplasia or osteonecrosis has been reported^37^, ^38^ In our study the expression of the gene/protein at E85 was significantly higher than that at E45, and the protein expression was more than 40 times higher, suggesting that G6P and Pi exchange was active and metabolism was enhanced in E85 condylar cells. ACP5 is involved in the production of reactive oxygen species, normal bone development, osteoblast regulation and macrophage function.^39^ Medical studies have found that mutations in ACP5 can cause metaphyseal dysplasia, autoimmune regulation disorders and nerve damage.^40^, ^41^, ^42^ However, the role of ACP5 in TMJ osteogenesis has not been reported. The role of ATP6V0d2 in osteoclast formation has been studied. It is not only regulates cell fusion in osteoclast differentiation, but also plays an important role in osteoclast specific proton pump mediated by bone resorption and extracellular acidification.^43^ The upregulation of Atp6v0d2 is beneficial to the formation of osteoclasts. In line with these findings, mice with ATP6V0d2 gene deletion showed increased bone mass, but knockout of ATP6V0d2 gene in ovariectomized mice did not inhibit the occurrence of osteoporosis.^44^ In our study it was found that the expression of SLC37A2, Acp5 and ATP6V0d2 at E85 were upregulated compared with E45, especially in the proteomic analysis, which showed that both SLC37A2 and Acp5 were upregulated more than 40‐fold, indicating that a large number of osteoclasts were produced compared with E45. The osteoblasts and osteoclasts were associated with each other to maintain the dynamic balance of bone metabolism.

Studies on the molecular regulation mechanism of TMJ condyle ossification in large mammal embryos have not been reported. Here we report a preliminary study of the key genes regulating condyle ossification of TMJ in pig embryo. In this study, 15 differential genes associated with bone development, regeneration and pathogenesis of osteoarthritis through literature survey were selected for QPCR verification. The other 22 genes were not verified by QPCR, but the future study based on the validation results for the 15 genes will verify other genes. In follow‐up studies, all the 37 differential genes will be verified by QPCR, immunohistochemistry and western blot in TMJ of minipigs of different gestational ages. Insufficient sample size of embryos at E45 and E85 is another limitation of this study. In future research, our research group will expand the sample size to exclude differences between different litters. We believe that with further in‐depth studies, the discovery of molecules regulating the ossification of the TMJ condyle during the embryonic period will provide a basis for the treatment of temporomandibular joint diseases and tissue engineering research.

## AUTHOR CONTRIBUTIONS

**Lei Xiang** and **Yongfeng Li**: conceptualization, investigation, methodology, visualization, writing of original draft, and reviewing and editing the manuscript. **Xuewen Wang**, **Huawei Liu**, and **Ping Chang**: conceptualization, investigation, methodology validation, and writing, reviewing, and editing the manuscript. **Xiaodan Mu** and **Tengyue Tianteng**: formal analysis, investigation, and writing, reviewing, and editing the manuscript. **Min Hu**: conceptualization, project administration, obtaining resources, and writing, review, and editing the manuscript.

## FUNDING INFORMATION

This research was funded by the National Key Research and Development Program of China (2017YFB1104103) and Beijing Municipal Health Commission (BJRITO‐RDP‐2023).

## CONFLICT OF INTEREST STATEMENT

The authors have no potential conflicts of interest to declare with respect to the research or the authorship and/or publication of this article.

## EHICS STATEMENT

This study does not contain any studies with human subjects. It was approved and supervised by the Institutional Animal Care and Use Committee of Chinese PLA General Hospital (Beijing, China; approval document no. 2020‐X16‐109).
